# An interpretable transformer network for the retinal disease classification using optical coherence tomography

**DOI:** 10.1038/s41598-023-30853-z

**Published:** 2023-03-03

**Authors:** Jingzhen He, Junxia Wang, Zeyu Han, Jun Ma, Chongjing Wang, Meng Qi

**Affiliations:** 1 Department of Radiology, Qilu Hospital of Shandong University, Jinan, 250012 China; 2 School of Information Science and Engineering, Shandong Normal University, Jinan, 250358 China; 3 School of Mathematics and Statistics, Shandong University, Weihai, 264209 China; 4 School of Cyber Science and Engineering, Southeast University, Nanjing, 211189 China; 5 China Academy of Information and Communications Technology, Beijing, 100191 China

**Keywords:** Biomedical engineering, Diseases

## Abstract

Retinal illnesses such as age-related macular degeneration and diabetic macular edema will lead to irreversible blindness. With optical coherence tomography (OCT), doctors are able to see cross-sections of the retinal layers and provide patients with a diagnosis. Manual reading of OCT images is time-consuming, labor-intensive and even error-prone. Computer-aided diagnosis algorithms improve efficiency by automatically analyzing and diagnosing retinal OCT images. However, the accuracy and interpretability of these algorithms can be further improved through effective feature extraction, loss optimization and visualization analysis. In this paper, we propose an interpretable Swin-Poly Transformer network for performing automatically retinal OCT image classification. By shifting the window partition, the Swin-Poly Transformer constructs connections between neighboring non-overlapping windows in the previous layer and thus has the flexibility to model multi-scale features. Besides, the Swin-Poly Transformer modifies the importance of polynomial bases to refine cross entropy for better retinal OCT image classification. In addition, the proposed method also provides confidence score maps, assisting medical practitioners to understand the models’ decision-making process. Experiments in OCT2017 and OCT-C8 reveal that the proposed method outperforms both the convolutional neural network approach and ViT, with an accuracy of 99.80% and an AUC of 99.99%.

## Introduction

The number of patients suffering from retinal illness has increased dramatically in recent years^1,2^. Age-related macular degeneration (AMD) and diabetic macular edema (DME) are two frequent retinal disorders that can lead to lifelong blindness. AMD, which comes in two forms: dry AMD and wet AMD, is the most prevalent cause of blindness in people over 65. Patients with dry AMD present drusen on the retina, and most patients with wet AMD show choroidal neovascularization (CNV)^3^. DME is a diabetic complication that causes structural alterations in the retinal neurovascular systems, resulting in visual loss^4^. It is caused by a rupture in the retinal vessel walls, which results in the accumulation of fluid and proteins in the retina^5^. According to survey statistics, about 25% of diabetic retinopathy patients develop to DME^6^. With early identification and treatment, the course of fundus disease can be delayed.

Optical coherence tomography (OCT) is a sophisticated ophthalmic imaging technique to display the cross-section of retina layers. It has the advantages of being non-contact, non-invasive, and rapid imaging^7^. Ophthalmologists regard OCT as one of the most important tools for the quantification, analysis, and treatment design of retinal diseases. However, there are certain difficulties in manually diagnosing retinal OCT images. First, as the number of patients grows year by year, relying solely on qualified medical professionals to make diagnoses will no longer be sufficient to meet the diagnostic and therapeutic requirements^8^. Second, the characteristics of certain lesions are not readily obvious, leading to misinterpretation and missed diagnoses. Moreover, a large number of patients have gone undiagnosed in the early stages of the disease due to a lack of medical care in some locations, which will cause disease aggravations.

Computer-aided diagnosis (CAD) is an effective method to address these problems. Although some breakthroughs have been obtained in the field of classification of retinal OCT images, there are some challenges of design well-performing machine learning CAD systems, such as complicated feature selection and high computational cost. In recent years, deep learning has developed rapidly and has shown brilliant performance in the field of computer vision. Deep learning has become the mainstream algorithm for retinal OCT image classification. It uses convolutional neural layers to automatically learn image features from low level to high level, which overcomes the shortcomings of manual feature extraction.

Several scholars have explored the application of convolutional neural networks (CNNs) for the automatic diagnosis of OCT images. Perdomo et al.^9^ developed an OCT-Net to classify normal retina and three common retinal diseases. The proposed network extracted and displayed information that was interpretable for clinical diagnosis. Kamran et al.^10^ proposed a retinal disease classification framework consisting of two joint networks, which combine supervised and unsupervised approaches to improve the robustness and accuracy of identifying retinal diseases. In addition, Rajagopalan et al.^11^ trained a deep learning-based fully automatic diagnosis system and used the Kuan filter to remove speckle noise from the input image, which provided higher classification accuracy for large public OCT datasets. Song et al.^12^ proposed a depth inference mechanism for the diagnosis of glaucoma, which combined OCT and visual field (VF) examination to effectively utilize complementary information from different modalities. jin et al.^13^ proposed to improve the performance and interpretability of traditional DL models by implementing segmentation based on prior human knowledge. Vidal et al.^14^ transforms binary masks into photorealistic OCT images using image-to-image generative adversarial networks. Based on the clinical relationship between retinal shape and the presence of DME fluid, this method generates pathological and non-pathological samples by changing the dichroic mask morphology. Previous works have shown that the deep learning method achieved a matching or exceeding performance to that of ophthalmologists with significant clinical experience^15,16^.

The evolution of network architectures in natural language processing (NLP) has promoted computer image processing from CNN to the sequence network Transformer. Vision Transformer (ViT) has become the most prevalent architecture in computer vision. Designed for sequence modeling and transduction activities, ViT is notable for its use of self-attention based on windows to model long-range dependencies in the whole image. Wen et al.^17^ recently applied the ViT framework to OCT images for auxiliary diagnosis of ocular abnormalities. They employed CNN to extract local features and the ViT to consider the image’s global information, resulting in an increase in overall accuracy, sensitivity, and specificity. Their proposed method illustrates the advantages of ViT for modeling global dependencies.

However, due to domain differences, converting the Transformer from natural language processing (NLP) to computer vision presents two obstacles. On the one hand, ViT cannot capture features at multiple scales, because the language is not affected by scale changes. On the other hand, image pixels have a larger resolution than text words, resulting in an exponential rise in computation. Fortunately, Liu et al.^18^ introduced the Swin Transformer, a hierarchical vision transformer that increased computational efficiency by using a shifted-window strategy. They also developed a Patch Merging method for flexibly synthesizing small patches into large patches, thereby widening the perceptual field and providing feature information on multiple scales.

**Figure 1:**
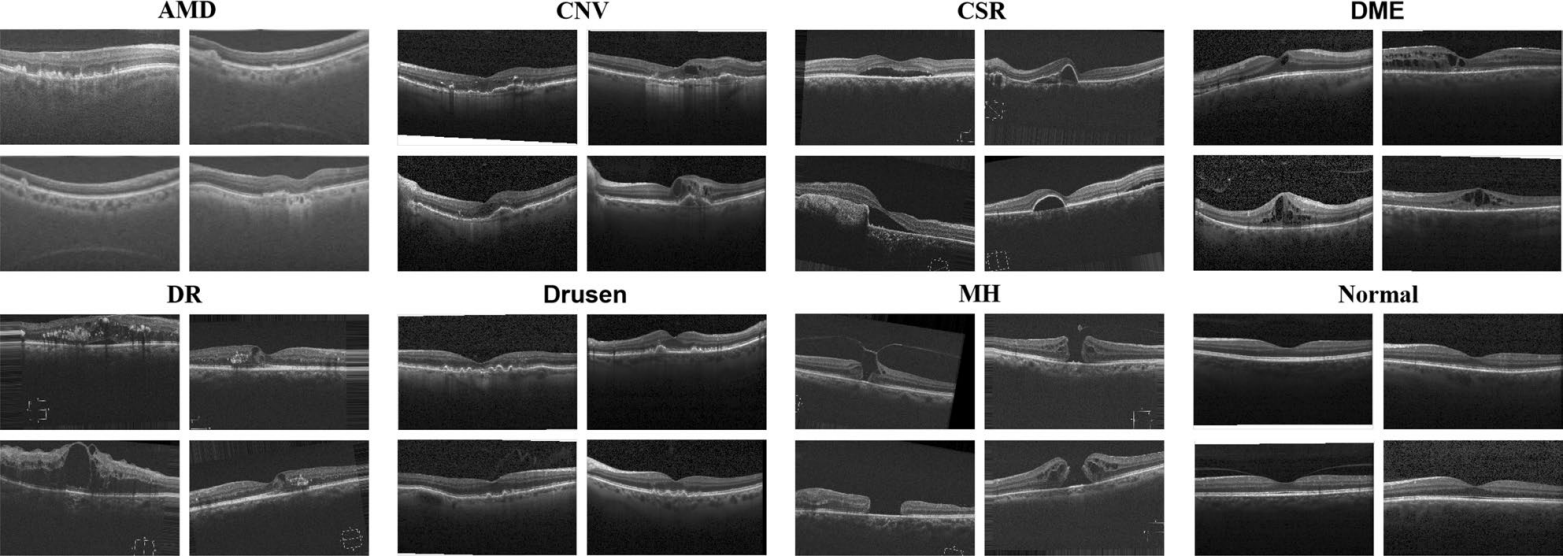
Examples of OCT images in eight classes, including AMD, CNV, CSR, DME, DR, drusen, MH and Normal.

In this paper, inspired by the Swin Transformer, we proposed an automatic diagnosis network Swin-poly Transformer for classifying OCT images into different categories. Figure 1 depicts examples from eight categories of fundus diseases. Furthermore, We adopt the PolyLoss as a loss function, which adjusts polynomial coefficients automatically for better retinal OCT image classification. In addition, the visual interpretation method is adopted in the inference stage to improve the model’s interpretability. We utilize the post-hoc interpretation method Score-CAM^19^ to generate confidence score maps, which highlight the discriminative features and thereby assist clinicians to understand the model’s decision-making.

In summary, the contributions of this work are as follows:

- In this paper, We propose the Swin-poly Transformer that combines the multi-scale features and the Poly loss to improve the performance of automatic retinal OCT classification.
- To intuitively understand the suggested model decision, we generate a heatmap based on the Score-CAM and apply it to the original image to highlight the tumor region.
- The suggested strategy achieves state-of-the-art performance in OCT2017, outperforming both the ViT network approach and convolutional neural network approach, with an accuracy of 99.80% and an Area Under Curve of 99.99%.

## Related work

Several studies have looked into using deep learning algorithms to identify OCT images. Lu et al.^15^ and Bhadra et al.^20^ trained a deep multi-layered CNN to classify OCT images into healthy, dry AMD, wet AMD, and DME categories. Kermany et al.^21,22^ and ^23^ investigated the application of deep transfer learning for the automatic diagnosis of diabetic retinopathy in OCT images. Das et al.^24^ introduced a multi-scale deep feature fusion (MDFF) network to contribute discriminative features and complementary information to the classifier. Huang et al.^25^ suggested a layer-guided CNN (LGCNN) for identifying normal retina and three common types of macular pathologies (CNV, DME and Druse). It employed an effective segmentation network to build retinal layer segmentation maps and then integrate the information from two lesion-related layers to improve OCT classification. Kim and Tran^26^ implemented a CNN-based ensemble learning model through several CNNs to further improve classification performance. Similarly, Alqudah et al.^27^ trained a CNN classification model on a large number of OCT images for distinguishing five types of retinal diseases, which achieved an overall accuracy of 0.953.

Recently, there have been new advances in OCT image classification based on deep learning. Saleh et al.^28^ and Subramanian et al.^29^ explored the transfer learning of pre-trained CNN networks to diagnose retinal disorders. The accuracy and robustness of transfer learning with CNN for retinal disease classification is demonstrated by comparison with other classifiers and human experts. Wen et al.^17^ proposed a lesion-localization convolution transformer (LLCT) network. It combines both convolution and self-attention to classify ophthalmic diseases and localize the retinal lesions. This design takes advantage of CNN’s extracting local features and the transformer’s consideration of global context and dynamic attention, accurately classifying and localizing retinal lesions. In addition, Saleh et al.^30,31^ developed a multi-criteria decision platform to investigate how to evaluate diagnostic models for retinal diseases and to enable the decision model to select the appropriate diagnostic model. The platform uses an entropy technique with ideal solution similarity ranking and employed nine quantitative criteria to evaluate models, facilitating reliable and fast diagnosis. Karthik and Mahadevappa^32^ proposed a modern diagnosis system for OCT image classification. They replace the residual connection in three ResNet architectures with EdgeEn block and cross-activation for increasing the contrast of the derivatives to generate sharper features, successfully increasing the classification accuracy. In this work, we propose to employ a transformer network that combines the multi-scale features and the Poly loss to improve the performance of automatic retinal OCT classification.

## Materials and methods

### Materials

We use the retinal OCT image datasets OCT2017^21^ and OCT-C8^33^ to evaluate the proposed method. We follow the original data division strategy and use the handout method to split the training, validation set, and test sets. The first dataset consists of 109,312 images, where 108,312 images are used for training, 32 for validation, and 968 for testing. In the training set, there are 37,205 retinal OCT images with CNV, 11,348 images with DME, 8616 images with drusen, and 26,315 normal images in the training set. In the validation and test sets, 8 and 242 OCT images were included in each category, respectively. The second dataset OCT-C8 consists of 24,000 images and is divided into eight categories: Age-related macular degeneration (AMD), Choroidal Neovascularisation (CNV), Diabetic macular edema (DME), Drusen, Macular Hole (MH), Diabetic Retinopathy (DR), Central Serous Retinopathy (CSR) and one for healthy classes. Where 25,600 images are used for training, 2800 for validation, and 2800 for testing. Each category includes 3200 for training and 350 for validation and testing respectively.

Data preprocessing and augmentation are performed prior to model training. Deep learning models are a data-driven way to learn task-related features. These models are based on the assumption that training data and test data have the same distribution. In a real scenario, this hypothesis holds only when the sample size is large enough. However, collecting numerous labeled medical images is difficult compared to natural images^1^ because labeling medical images requires a lot of time and effort from experienced experts. Numerous works have proven that data augmentation is an effective method to improve the diversity of training data, which contributes to enhancing the generalization and stability of the model^34^. Additionally, the features’ scale and rotation invariance are not captured by the CNN model. Therefore, data augmentation methods, including random rotation, flipping and mirroring, are adopted to increase the diversity of training images. Furthermore, to match the input of the model, all images are resized to $224 \times 224$ and normalized to [0, 1]. Finally, converting data into tensors and sending them to the proposed model.

### Overall framework

**Figure 2:**
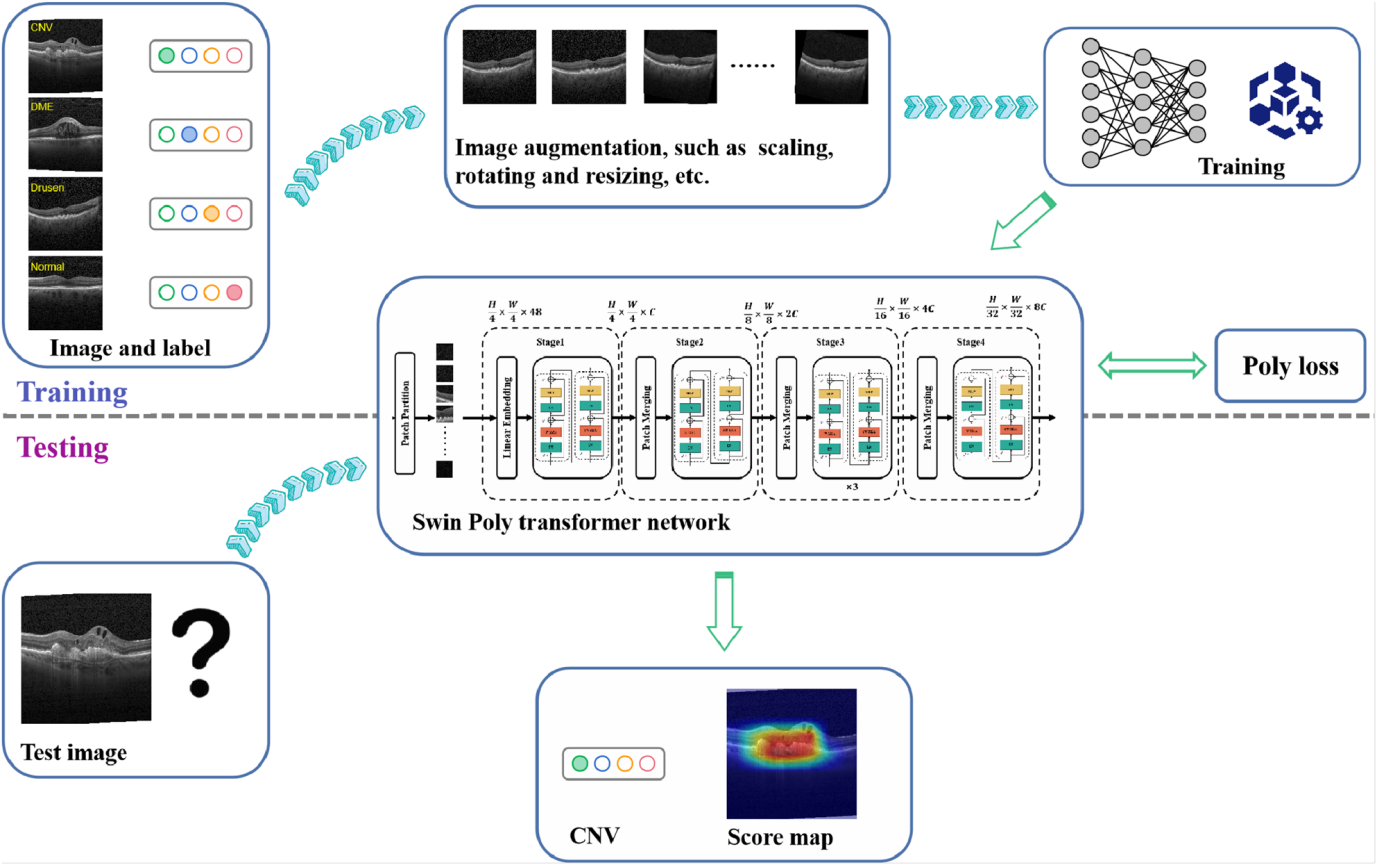
The overall framework of the proposed method.

We present a Swin-Poly Transformer network, which combines Swin Transformer^18^ and PolyLoss, for the automatic diagnosis of retina diseases in OCT images. Moreover, the proposed method provides visual interpretation based on the score-CAM method. The pipeline of the proposed method is depicted in Fig. 2. Specifically, in the training stage, random data augmentation is performed on the training set to improve the generalization ability of the model. After that, the enhanced images are fed into Swin Transformer in batches for weights and parameters learning. Furthermore, PolyLoss is employed in this work to automatically adjust polynomial coefficients for better retinal OCT image classification. Based on the prediction, score-CAM generates a visual explanation to help understand the model’s decision-making.

### Swin Transformer for multi-scale feature representation

The Transformer architecture and its adaptation on image classification^35^ performs global self-attention by establishing a relationship between one token and all others. However, in contrast to convolutional neural networks, induction biases, i.e., two-dimensional neighborhood structure (locality) and translational equivalence, are lost in Vit^18^. Specifically, the two neighborhood structure describes the neighboring regions with similar features in an image. Translational equivalence means that objectives in an image should get the same result (labels) no matter where they are moved. Scholars have demonstrated that the lack of inductive bias breaks down when the amount of data is large enough^36^. However, access to millions of labeled medical images is difficult due to privacy and ethical requirements. Moreover, the pixel resolution in images is much higher than the length of words in text paragraphs, resulting in an increase in the amount of computation. Therefore, in this work, we investigate the use of the Swin Transformer to express the multi-scale feature representation in OCT images. It can reduce the computational complexity of self-attention by exploiting the prior knowledge of induction bias in ViT.

#### Architecture of Swin Transformer

**Figure 3:**
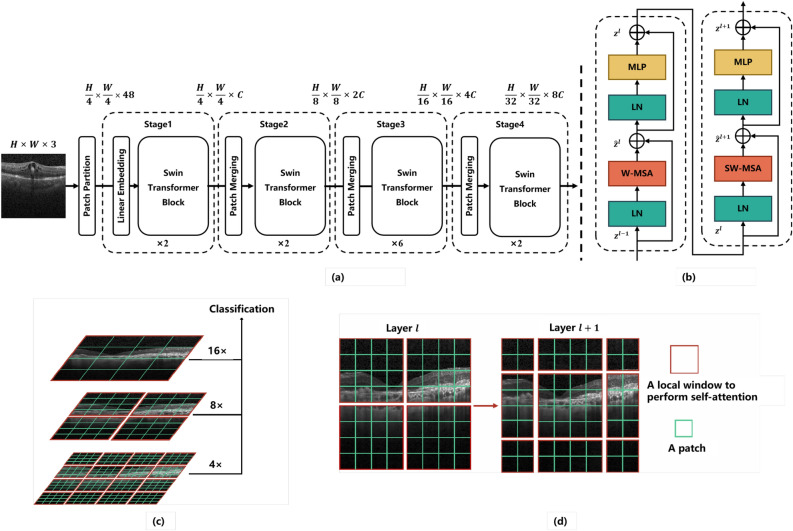
(**a**) The overall architecture of Swin Transformer, which is adapted from Liu et al.^18^. (**b**) Two successive Swin Transformer blocks. (**c**) The hierarchical structure of Swin Transformer for extracting multi-scale feature representation. (**d**) An illustration of the shifted window strategy for computing self-attention in the Swin Transformer architecture.

An overview of the Swin Transformer is presented in Fig. 3. A patch partition module first splits an input image of $224 \times 224$ into non-overlapping patches of size $4 \times 4$. Each patch is treated as a ”token”, and the patch tokens are projected to the *C* dimension using a linear embedding layer. Following that, two successive Swin Transformer blocks with self-attention computation are applied to these patch tokens to control the number of tokens, as shown in Fig. 3b. A ”stage” is the combination of the linear embedding layer and the Swin Transformer blocks. The design of the Swin Transformer is similar to the layer structure of a CNN, where the resolution of each stage is halved and the number of channels is doubled. To produce hierarchical representations, the Swin-Transformer reduces the number of tokens by merging patch layers as the network gets deeper. An example of hierarchical representation is illustrated in Fig. 3c.

#### Swin Transformer block

There are two units in the Swin Transformer block. Each unit consists of two normalization layers (LayerNorm), a self-attention module, and a multilayer perceptron (MLP) layer. In the Swin Transformer block, the standard multi-head self attention (MSA) module in ViT is replaced with two successive Swin Transformer modules, the window multi-head self attention (W-MSA) module and shifted window multi-head self attention (SW-MSA) module, as illustrated in Fig. 3b. Each unit consists of two normalization layers (LayerNorm), a self-attention module, and an MLP layer. The first unit uses the Window MSA (W-MSA) module, while the second unit uses the shifted Window MSA (SW-MSA) module. LayerNorm layers are added before each MSA module and each MLP layer, and the residual connection is employed after each module.

The Swin Transformer conducts self-attention on windows to reduce computational complexity. While in ViT, standard MSA is used for global attention. The relationship between each patch is computed based on all other patches. However, the computational complexity is quadratic because of the enormous number of patches, making it unsuitable for high-resolution images. For effective modeling, Swin Transformer uses the W-MSA for calculating self-attention within a local window. Where a window is a set of patches that uniformly and non-overlappingly split the entire image. Assuming that each window contains $M \times M$ patches, the computational complexities of the global MSA module and W-MSA in an image of $h \times w$ patches are as follows.

1 $$\begin{aligned} \Omega (MSA)= & {} 4hwC^2 + 2(hw)^2C \end{aligned}$$

2 $$\begin{aligned} \Omega (W-MSA)= & {} 4hwC^2 + 2M^2hwC \end{aligned}$$

where $h \times w$ represents the number of patches in whole images, and *C* is the channel of patches channel. In Eq. (1), the complexity is quadratic to patch number $h \times w$. While in Eq. (2), the complexity of the latter is linear when *M* is fixed (set to 7 by default). For a large $h \times w$, global self-attention computation is generally unaffordable, whereas window-based self-attention is scalable.

#### Shifted window for self-attention

However, the window-based self-attention (W-MSA) lacks cross-window connections, which limits the model’s modeling capabilities. In order to introduce the cross-window connection while maintaining efficient computation of non-overlapping windows, a shift window partitioning method is proposed in the Swin Transformer block. Figure 3d illustrates the shifted window partitioning strategy. In the *l*-th layer of the Swin Transformer, we use the window partitioning strategy for calculating the local attention. The $8 \times 8$ feature map is uniformly divided into $2 \times 2$ windows of size $4 \times 4$ ($M = 4$). Then, the next layer $l+1$ adopts the window partitioning configuration from the front layer to generate new windows, by replacing the window $\left( \left\lfloor \frac{M}{2}\right\rfloor , \left\lfloor \frac{M}{2}\right\rfloor \right)$ pixels from the regular partitioned window. The self-attention computation of the new window crosses the boundary of the previous window in layer *l*, providing a connection between them. By using the shifted window partitioning strategy, the successive Swin Transformer blocks are calculated as:

3 $$\begin{aligned} {\hat{z}} ^ l&= \mathrm {W-MSA} \left( LN \left( z^{l-1}\right) \right) +z^{l-1} \end{aligned}$$

4 $$\begin{aligned} z ^ l&= MLP \left( LN \left( {\hat{z}}^{l}\right) \right) +{\hat{z}}^{l}, \end{aligned}$$

5 $$\begin{aligned} {\hat{z}} ^ {l+1}&= \mathrm {SW-MSA}\left( LN \left( z^{l}\right) \right) +z^{l}, \end{aligned}$$

6 $$\begin{aligned} z ^ {l+1}&= MLP \left( LN \left( {\hat{z}}^{l+1}\right) \right) +{\hat{z}}^{l+1} \end{aligned}$$

where ${\hat{z}}^l$ and $z^l$ represent the output features of the W-MSA module and MLP in the *l* layer, ${\hat{z}}^l$ and $z^l$ represent the output features of the W-MSA module and MLP in the *l* layer. The shift window partitioning method introduces the connection between adjacent non-overlapping windows in the previous layer, which helps to establish the relationship of the model.

**Figure 4:**
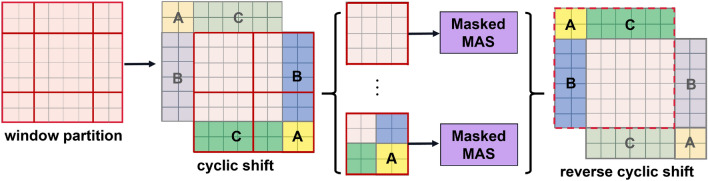
Efficient batch computation approach for self-attention in shifted window partitioning.

The window partitioning strategy produces multiple new windows of different sizes, and some of the new windows are smaller than $M \times M$. To calculate self-attention, one typical method is to fill all windows into $M \times M$. This method, however, will result in a rise in the number of windows. As shown in Fig. 3d, the number of windows increases from $2 \times 2$ to $3\times 3$ after the window transformation strategy, which obviously increases the calculation cost of the model. To alleviate this problem, Swin Transformer proposes an efficient batch computation approach of cyclic shifting toward the top-left direction, as illustrated in Fig. 4. After shifting, the window computed in batches may consist of several windows in the feature map that are not adjacent to each other. Therefore, to confine the calculation of self-attention to each sub-window, a masking method is applied. With the cyclic shift, the number of batch windows remains the same as the number of regular window divisions, thus improving computational efficiency.

### Loss function

In this paper, PolyLoss is used to optimize the OCT classification model. PolyLoss is proposed by Leng et al.^37^, which provides a framework for understanding and refining the commonly used cross-entropy loss. It allows the importance of multiple polynomial bases to be easily modified based on the targeting tasks and datasets. As a result, we use the PolyLoss in this study to automatically change polynomial coefficients for better retinal OCT image classification.

Applying the Taylor expansion, the cross entropy loss in the bases of $(1 - P_{t})^j$ can be decomposed as

7 $$\begin{aligned} L_{Poly} = - log(P_{t}) = \sum _{j=1}^{\infty } 1/j(1 - P_{t})^{j}=(1 - P_{t}) + 1/2(1 - P_{t}) + \cdots \end{aligned}$$

The Eq. (7) can be further condensed in the form of $\sum _{j=1}^{\infty } \alpha _{j}(1-P_{t})^{j}$, where $\alpha _{j}\in {\mathbb {R}}^{+}$ is the polynomial coefficient and $P_{t}$ is the prediction probability of the target category label. Each polynomial base $(1-P_{t})^j$ is weighted by a corresponding polynomial coefficient $\alpha _{j}$, allowing us to easily adjust the importance of different bases for various applications. The PolyLoss is equivalent to the cross-entropy loss when $\alpha _{j} = 1/j$ for all *j*.

Leng et al., propose perturbing the leading polynomial coefficients in cross-entropy to reduce the number of $\alpha _{j}$. They substitute the $j-th$ polynomial coefficient in cross entropy loss 1/*j* with $1/j + \varepsilon _{j}$.

8 $$\begin{aligned} L_{Poly}= & {} \underbrace{(\varepsilon _1+1)(1-P_t)+ \cdots +(\varepsilon _N + 1/N)(1-P_t)^N }_{perturbed\ by\ \varepsilon _j} + \underbrace{1/(N+1)(1-P_t)^{N+1} + \cdots }_{same\ as\ L_{CE}} \end{aligned}$$

9 $$\begin{aligned}= & {} - \,\,log (P_t) + \sum _{j=1}^{N}\varepsilon _{j} (1-P_{t})^j \end{aligned}$$

where $j \in [-1/j, \infty )$. *N* is the number of leading term coefficients to be tuned. PolyLoss experiments found that tuning the first polynomial term yields the largest significant gain. As a result, the Eq. (8) can be reduced to:

10 $$\begin{aligned} L_{Poly} = - \,\, log (P_t) + \varepsilon _{1}(1 - P_{t}) \end{aligned}$$

In this paper, we set $\varepsilon _{1} = 2$ following the configuration on ImageNet image classification.

### Score-CAM for visual interpretation

Although deep learning has been widely applied in a variety of scenarios such as medical image analysis and consultation assistance, the majority of existing deep learning networks are black box models with low interpretability. However, medical applications have a great demand for the interpretability of deep learning models due to the involvement of ethics and life health. Therefore, decisions regarding artificial intelligence applications should be supported by rationales and explanations. Some scholars have proposed post-hoc methods to explain the predicted behavior after the training is completed, such as Saliency Maps^38^, guided backpropagation (GuidedBP)^39^ and class activation mapping (CAM)^40^. In this work, we introduce Score-CAM, a robust and reliable interpretation method, to provide a fair interpretation of the decision process. Score-CAM treats the importance of features as a function of the confidence level, thus getting rid of the dependence on gradients.

**Definition: Increase of confidence** Given a general function $Y = f(X)$ that takes an input vector $X = [x_0, x_1, \ldots , x_n]^ \top$ and outputs a scalar *Y*. For a known baseline input $X_b$, the contribution $c_i$ of $x_i, (i \in n - 1])$ towards *Y* is the change of the output by replacing the $i-th$ entry in $X_b$ with $x_i$. Formally,

11 $$\begin{aligned} c_i = f(X_b \circ H_i) - f(X_b) \end{aligned}$$

where *Hi* is a vector with the same shape of $X_b$ but for each entry $h_j$ in $H_i$, $h_j = {\mathbb {I}}[i = j]$ and $\circ$ denotes Hadamard Product.

We define the trained Swin Transformer as $Y = f(X)$ that outputs a class probability scalar *Y*. We pick the second normalization layer in the last Swin Transformer block and the corresponding activation as *A*. Denote the *k*th channel of activation *A* as $A_{k}$. Therefore, the contribution score $A_{k}$ towards *Y* is defined as

12 $$\begin{aligned} C(A_{k}) = f(X \circ H_{k}) - f(X_b) \end{aligned}$$

where

13 $$\begin{aligned} H_{k} = s(Up(A_{k})) \end{aligned}$$

$Up(\cdot )$ represents the operation that upsamples $A_{k}$ into the input size. In this way, each upsampled activation map not only presents the most relevant spatial location to the internal activation map but also can be used directly as a mask to disturb the input image. $s(\cdot )$ is a normalization function that maps each element in the activation map matrix into [0, 1], which generates a smoother mask $H_{k}$. The normalization function $s(\cdot )$ is represented as

14 $$\begin{aligned} s(A_{k}) = \frac{A_{k}- min A_{k}}{max A_{k} - min A_{k}} \end{aligned}$$

Then, the final visualization is obtained by a linear combination of weights and activation mappings. In addition, ReLU is also applied to the linear combination of mappings, since we are only interested in those features that have a positive impact on the category of interest.

15 $$\begin{aligned} V_{Score-CAM} = ReLU \left( \sum _k \alpha _k^c A_l^k \right) \end{aligned}$$

Finally, we show the visualization in the form of heatmap and apply it to the input image for explaining the decision process.

### Implement details

The experiments are conducted on Linux Ubuntu 16.04, Python 3.6, and Pytorch 1.11.0. Models are trained on an NVIDIA Tesla V100 GPU. We initialize the weights with Xavier initialization^41^ and optimize them during training with the Adam optimizer using $\beta _{1}=0.900$. The initial learning rate is $2e^{-4}$ and then decays into $1e^{-5}$ lastly. All of the OCT images are resized to $224 \times 224$. The batch size was set to 32. We train each model for 200 epochs. The model at the last epoch is used to evaluate performance. Moreover, for the dataset OCT2017, we adopt the weight loss strategy to alleviate the incorrect prediction caused by class imbalance.

### Evaluation of classification models

For evaluating the classification performance, we apply the softmax method to convert logits into class probabilities, and then take the highest probability value as the predicted category. Accuracy, precision, recall, and F1-score are used as evaluation metrics. The formulas of evaluation metrics are as follows.

16 $$\begin{aligned} Accuracy= & {} \frac{TP+TN}{TP+FP+TN+FN} \end{aligned}$$

17 $$\begin{aligned} Precision= & {} \frac{TP}{TP+FP} \end{aligned}$$

18 $$\begin{aligned} Recall= & {} \frac{TP}{TP+FN} \end{aligned}$$

19 $$\begin{aligned} F1-score= & {} 2\cdot \frac{precision \cdot recall}{precision + recall} \end{aligned}$$

Where TP, TN, FP, and FN represent the number of true positives, true negatives, false positives, and false negatives, respectively. For the four classes OCT classification, TP is defined as the number of cases correctly identified as a category, TN as the number of negative cases correctly identified as a negative class by the model, FP as the number of negative samples incorrectly identified as positive classes, and FN as the number of positive cases incorrectly identified as negative categories. In addition, the area under curve (AUC) is an additional metric for further evaluate the proposed method. The larger the AUC, the closer the prediction is to the true label.

**Table 1:** Experimental results on OCT image classification.

Dataset	Method	Class	Accuracy	Precision	Recall	F1-Score	AUC
OCT2017	LLCT^17^	CNV	0.9810	0.9350	0.9940	0.9760	0.9960
		DME	0.9960	0.9860	0.9960	0.9950	0.9970
		Drusen	0.9810	0.9960	0.9280	0.9990	0.9190
		Normal	0.9960	0.9960	0.9880	0.9990	0.9590
		Average	0.9885	0.9783	0.9765	0.9923	0.9678
	ViT	CNV	0.9800	0.9878	1.0000	0.9938	0.9993
		DME	0.9880	0.9918	0.9959	0.9938	0.9990
		Drusen	0.9920	0.9917	0.9876	0.9897	0.9985
		Normal	1.0000	0.9916	0.9793	0.9855	0.9999
		Average	0.9900	0.9907	0.9907	0.9907	0.9992
	Swin Transformer	CNV	1.0000	0.9881	1.0000	0.9940	0.9999
		DME	1.0000	1.0000	1.0000	1.0000	0.9995
		Drusen	0.9880	1.0000	0.9880	0.9940	0.9998
		Normal	1.0000	1.0000	1.0000	1.0000	1.0000
		Average	0.9970	0.9970	0.9970	0.9970	0.9998
	Ours	CNV	1.0000	0.9960	1.0000	0.9980	1.0000
		DME	0.9960	1.0000	0.9960	0.9980	0.9996
		Drusen	1.0000	0.9960	1.0000	0.9980	1.0000
		Normal	0.9960	1.0000	0.9960	0.9980	1.0000
		Average	**0.9980**	**0.9980**	**0.9980**	**0.9980**	**0.9999**
OCT-C8	Vit	AMD	1.0000	0.9972	1.0000	0.9886	1.0000
		CNV	0.8657	0.8511	0.8657	0.8584	0.9845
		CSR	0.9886	0.9971	0.9943	0.9957	0.9999
		DME	0.7771	0.8576	0.7743	0.8138	0.9720
		DR	0.9971	0.9886	0.9886	0.9886	0.9991
		Drusen	0.7429	0.7424	0.7000	0.7206	0.9543
		MH	0.9914	0.9915	0.9943	0.9929	0.9995
		Normal	0.7571	0.7053	0.8000	0.7497	0.9686
		Average	0.8896	0.8913	0.8896	0.8898	0.9847
	Swin-Vit	AMD	1.0000	1.0000	1.0000	1.0000	1.0000
		CNV	0.8516	0.8493	0.9657	0.9037	0.9947
		CSR	0.9821	1.0000	0.9971	0.9986	1.0000
		DME	0.9122	0.9324	0.9057	0.9188	0.9933
		DR	0.9836	0.9859	1.0000	0.9929	1.0000
		Drusen	0.9327	0.9708	0.76	0.8526	0.9882
		MH	0.9812	1.0000	0.9886	0.9943	1.0000
		Normal	0.9257	0.8583	0.9514	0.9024	0.9954
		Average	0.9461	0.9496	0.9461	0.9454	0.9965
	Ours	AMD	1.0000	1.0000	1.0000	1.0000	1.0000
		CNV	0.9489	0.9389	0.9571	0.9477	0.9937
		CSR	1.0000	1.0000	1.0000	1.0000	1.0000
		DME	0.9439	0.9512	0.9457	0.9484	0.9919
		DR	1.0000	0.9972	1.0000	0.9986	0.9999
		Drusen	0.9200	0.9580	0.9114	0.9341	0.9888
		MH	1.0000	1.0000	0.9971	0.9986	0.9998
		Normal	0.9563	0.9254	0.9571	0.9410	0.9958
		Average	0.9711	0.9713	0.9711	0.9710	0.9962

Significant values are in [bold].

## Results

### Results on each category

In order to observe micro performance, we report the performance of several networks across each category of OCT2017 and OCT-C8. Table 1 shows the performance of LLCT^17^, Vision Transformer (ViT), Swin Transformer and our method. For dataset OCT2017, we observe that ViT outperforms LLCT in our setting, demonstrating the effectiveness of ViT for the task of OCT image classification. In addition, the performance on CNV and drusen images is further improved when Swin Transformer is used, which means that hierarchical multi-scale features contribute to better predictions. Swin Transformer obtained 1.0000 on four metrics (accuracy, precision, recall and F1 score) for DME and normal images, demonstrating the model’s ability to identify DME and normal images. Moreover, the PolyLoss leads to a further increase in classification accuracy, recall, F1-score, and AUC. The suggested method’s average accuracy, precision, recall F1-Score, and AUC are 0.9980, 0.9980, 0.9980, 0.9980, and 0.9999, respectively, slightly outperforming the LLCT’s 0.0095, 0.0197, 0.0215, 0.0057 and 0.0321. Although there is a small improvement in evaluation values, this improvement is visible in the dataset OCT2017, as all evaluation metrics are close to 1. The proposed Swin-Poly Transformer achieves the best performance on four metrics, suggesting the effectiveness of the proposed method. Similarly, we validate the proposed method on OCT-C8. For dataset OCT-C8, similarly, the proposed method surpasses ViT and Swin-VIT to achieve the best average performance. We find that Vit, Swin-Vit and our method all achieve high accuracy on AMD. The proposed method achieves performance close to 1 in the four categories of AMD, CSR, DR, and MH. Combining CNN with transformers offers a viable improvement direction for local and global feature fusion. All in all, the proposed method takes the best performance on average results.

We compare the floating-point operations per second (FLOPs), numbers of model parameters and inference time of VGG16, ViT, and our methods. The FLOPS of VGG16, ViT, and our methods are 15.4 G, 1.1 G and 4.5 G respectively. The Parameters of the three methods are 13.8 M, 22.1 M and 27.5 M. In the inference stage, predicting an image spend 2.72 ms, 5.9 ms and 12.6 ms. Although the inference time of our method is greater than that of VGG16 and ViT, for an OCT image, this speed is still satisfactory compared to manual reading.

**Table 2:** Performance of several deep learning networks for classification on OCT2017 and OCT-C8.

Dataset	Method	Accuracy	Recall	Precision	F1-score
Oct2017	Inception V3^42^	0.9660	0.9780	0.9740	0.9760
	Kermany et al.^21^	0.9610	0.9612	0.9610	0.9610
	Kaymak et al.^43^	0.9710	0.9960	–	–
	MDFF^24^	0.9960	0.9960	0.9960	0.9960
	LGCNN^25^	0.8990	–	–	–
	Islam et al.^23^	0.9860	–	0.9950	–
	Li et al.^22^	0.9860	0.9780	0.9940	0.9859
	Bhadra et al.^20^	0.9969	0.9969	0.9969	0.9968
	Kim et al.^26^	0.9890	0.9890	0.9960	0.9915
	Saleh et al.^28^	0.9850	0.9700	0.9700	0.9700
	LLCT^17^	0.9770	0.9770	0.9920	0.9844
	ViT^35^	0.9907	0.9907	0.9907	0.9907
	Swin Transformer^18^	0.9970	0.9970	0.9970	0.9970
	Ours	**0.9980**	**0.9980**	**0.9980**	**0.9980**
OCT-C8	Karthik et al.^32^ (ResNet34 based)	0.9240	0.9200	0.9300	0.9200
	Karthik et al.^32^ (ResNet50 based)	0.9030	0.9100	0.9100	0.9100
	Karthik et al.^32^ (ResNet101 based)	0.8450	0.8600	0.8600	0.8600
	Subramanian et al.^33^ (VGG16 based)	**0.9721**	**0.9725**	**0.9713**	**0.9725**
	ViT	0.8896	0.8913	0.8896	0.8898
	Swin-Transformer	0.9461	0.9496	0.9461	0.9454
	Ours	0.9712	0.9713	**0.9713**	0.9710

Significant values are in [bold].

**Figure 5:**
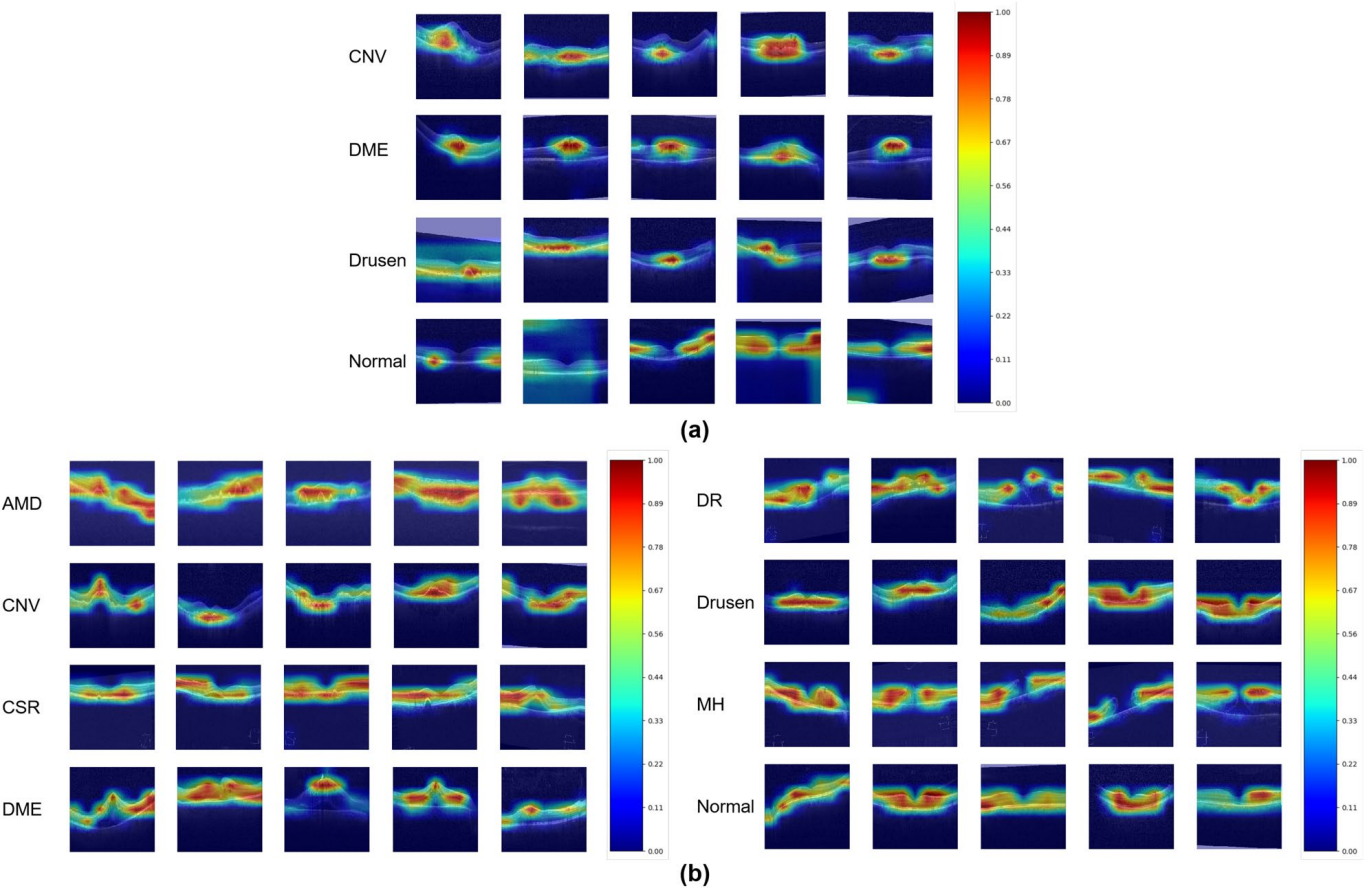
Confidence score maps on (**a**) OCT2017 and (**b**) OCT-C8 of proposed Swin-Poly Transformers.

**Figure 6:**
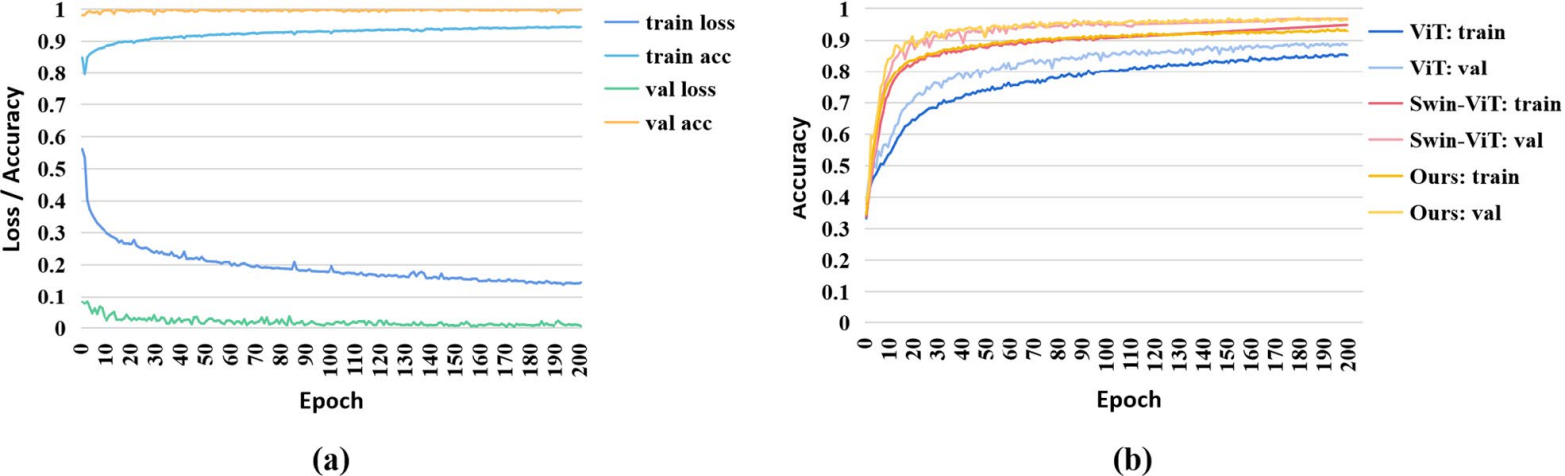
(**a**) The loss and accuracy curves of the proposed model in OCT2017. (**b**) The accuracy curves of different models on OCT-C8.

### Visualization

Further, we investigate the model decision-making mechanism in OCT2017. We use the post-hoc explanation approach Score-CAM^19^ to visualize the evidence of prediction. Score-CAM is a gradient-free visual interpretation method, where the importance of activation is encoded by the global contribution of the corresponding input instead of the local sensitivity (gradient information). We perform an interpretation experiment on 968 test images to see which regions contributed the most to the neural network’s prediction prognosis.

Figure 5 shows confidence score maps of the prediction results in OCT2017 and OCT-C8. The heat map highlights the regions that are connected with the target category. The redder the color, the higher the correlation with the predicted category. As can be seen in this figure, the score-CAM clearly shows the regions of interest. We notice that lesion regions are rendered as redder in the disease OCT images, for example, the first three rows of Fig. 5a and b right, Fig. 5b left, i.e., abnormal regions are given higher scores. In normal images (the last row of Fig. 5a and b right), the model pays more attention to the whole retina. These phenomena are consistent with clinical diagnosis, as ophthalmologists also identify diseases by looking at abnormal regions in OCT images.

## Discussion

We develop a Swin-Poly Transformer network to automatically and accurately identify retinal disease types. Using OCT images, we investigate the performance improvement of the Swin-Transformer model for retinal abnormality classification using multi-scale feature representation and loss optimization. Further, visual interpretation analysis is performed to determine whether the lesion areas of the model match the clinical diagnostic features.

In this paper, we compare the proposed method in dataset OCT2017, including ViT, Swin Transformer and Wen et al.^17^ in Table 1. ViT converts an image to several sequence tokens and then employs Multi-Head Self-Attention to model long-range dependencies between tokens. This structure considers the image’s global information, leading to an increase in overall accuracy, sensitivity, and specificity (Table 2). Specifically, Wen et al.^17^ use the customized feature maps generated by CNN as the input of the self-attention network, exploiting local details from the CNN and global contextual and dynamic attention from the Transformer. In our experimental setting, the overall F1-score values for ViT, Swin Transformer, and Swin-Poly Transformer are 0.9907, 0.9970, and 0.9980 respectively. The performance of the Swin Transformer outperforms the ViT because of the utilization of multi-scale features. Swin Transformer shifts the window partition and then builds connections between adjacent non-overlapping Windows, thus combining low-level and high-level features. Furthermore, the Poly loss further improves the performance by refining the cross-entropy loss using Taylor expansion. It modifies a large number of polynomial bases according to the specific task and dataset to regulate the relevance of each basis. In particular, the Swin-Poly Transformer shows an AUC value of 0.9999, demonstrating the effectiveness of the proposed method. Experiments show that the accurate diagnosis provided by the proposed Swin-Poly Transformer can contribute to precision medicine.

We further compare the average performance of the Swin-Poly Transformer and other algorithms, including CNN and Transformer-based networks. We explore the performance of CNNs in OCT2017 from multiple perspectives, including general training (Lu et al.^15^ and Bhadra et al.^20^), transfer learning (Kermany et al.^21^, Li at al.^22^ and Islam et al.^23^), multi-scale/layer-guided feature fusion (MDFF^24^ and LGCNN^25^), and ensemble learning (Kim and Tran^26^). All results are shown in Table 2. From Table 2, we find CNN networks^24,25,43^ are useful algorithms for OCT image classification, achieving satisfactory results in OCT2017. Among the CNN-based algorithms, Bhadra et al.^20^ achieve the best performance with an accuracy of 0.9969, a recall of 0.9969, a precision of 0.9969 and an F1-score of 0.9968. These phenomena prove that with enough samples, CNNs are able to capture the subtle differences in each category of fundus OCT images in real scenes^44^. For Transformer-based backbones, the Swin Transformer outperforms the ViT on four metrics, suggesting the effectiveness of extracting multi-scale features using a multi-scale hierarchical strategy. Finally, the proposed method achieves the best performance with accuracy, recall, and precision of 0.9980, which indicates that the combination of multi-scale features and Poly loss benefits the performance improvement. We show the loss and accuracy curves in Fig. 6a. In the figure, the training loss first decreases gradually and then reaches equilibrium, indicating that the Swin-Poly Transformer has been fitted on the training data.

Furthermore, we verify the effectiveness of the Swin-Poly Transformer on another dataset, OCT-C8. All results are shown in Table 2. The proposed Swin-Poly Transformer exceeds the three ResNet-based models proposed by Karthik et al^32^. Moreover, the proposed Swein-Poly transformer achieves comparable performance to Subramanian et al.^29^ and further improves the interpretability of the model. Particularly, the proposed method exceeds the classical ViT in four evaluation indexes respectively. In addition, the accuracy, recall, accuracy, and F1 scores of Swin-Transformer using vanilla were 0.9461, 0.9496, 0.9461, and 0.9454, respectively. The proposed Swin-Poly Transformer achieves an accuracy of 0.9712, a recall of 0.9713, a precision of 0.9713, and an F1-score of 0.9710, which are 2.52%, 1.17%, 2.49% and 2.56% higher than Swin Transformer, respectively. The performance of the proposed method on OCT-C8 proves that the Swin-Poly Transformer is an effective algorithm for OCT image recognition. We show the training and validation accuracy curves in Fig. 6b. It can be found in the figure that Swin Transformer converges faster than ViT. The proposed Swin-Poly Transformer and Swin Transformer have comparable performance on the validation set. Furthermore, the accuracy of the Swin-Poly Transformer is higher than that of the Swin Transformer on test data. Additionally, in the first 50 epochs, the accuracy curve of the Swin-Poly Transformer is smoother than Swin Transformer on the training set. These phenomena suggest that using Poly loss contributes to boosting the generalization and robustness.

Observing intermediate layers facilitates revealing learned features and understanding the mechanism of decision-making^45^. Vision interpretability is an evolving area with the potential to help the developer and medical participant better understand how models work and gain new insights into revealing predictive failures^46^. In this paper, the gradient-free interpretation method Score-CAM is used to visualize the region of interest. We discover that the suggested model highlights abnormal areas of the image. The confidence score map displays the region around the anomaly in addition to the lesion of interest, indicating that contextual information about the immediate environment may be useful for prediction. The model appears to focus on the entire retinal layer for normal images, demonstrating its flexibility in learning complicated and representative features. Overall, these visualization results are remarkable and intuitive, confirming that the proposed model can appropriately identify regions of interest.

In this work, we propose an effective Swin-Poly Transformer for identifying normal OCT images and retinal abnormities. The Swin-Poly Transformer network has the potential to transform the currently limited classification model into a more analytical and flexible system, combing radiographic imaging, biological data and clinical reports. These approaches contribute to augmenting other emerging technologies, such as liquid biopsy; providing complementary information to guide clinical decision-making. However, despite the promising progress, the challenge of effectively integrating these computer-assisted diagnostic tools into regular practice remains. Perhaps most pressing is the need for extensive data sharing to build large, well-labeled datasets to develop a robust and scalable model. In future work, on the one hand, we expect to utilize complementing information from several modalities to simulate real diagnostic scenarios by combining multi-tasking or collaborative learning. On the other hand, we believe that intra- and inter-institutional data sharing will encourage models to perform better in real situations.

## Data Availability

The dataset analyzed during the current study is available in the Kaggle at https://www.kaggle.com/paultimothymooney/kermany2018.
